# Increased Expression of Inflammatory Cytokines and Discogenic Neck Pain

**DOI:** 10.1111/os.13963

**Published:** 2023-12-14

**Authors:** Xinjian Kang, Man Qian, Tao Qin, Mingli Liu, Haiwei Xu, Baoshan Xu

**Affiliations:** ^1^ Graduate School Tianjin Medical University Tianjin China; ^2^ Department of Orthopedics Traditional Chinese Medicine Hospital of Qinhuangdao Qinhuangdao China; ^3^ Department of Refractive Surgery Qinhuangdao Aier Ophthalmic Hospital Qinhuangdao China; ^4^ Department of Imaging Qinhuangdao Worker's Hospital Qinhuangdao China; ^5^ Department of Minimally Invasive Spine Surgery Tianjin Hospital Tianjin China

**Keywords:** cytokines, intervertebral disc degeneration, low back pain, neck pain, pathogenesis

## Abstract

**Objective:**

Although neck pain has become a serious economic and social problem worldwide, the etiology remains poorly understood. The aim of current study is to explore the possible pathogenesis of discogenic neck pain by analyzing the relationship between inflammatory cytokines and discogenic neck pain and provide a valuable reference for the prevention and treatment of discogenic neck pain.

**Methods:**

A total of 111 cervical disc samples were collected between October 1, 2021, and October 1, 2022: 38 samples from the discogenic neck pain group, 41 samples from the symptomatic control group, and 32 samples from the normal control group. The concentration of nitric oxide (NO), interleukin (IL)‐1, interleukin (IL)‐6, and tumor necrosis factor alpha (TNF‐α) was determined using the enzyme‐linked immunosorbent assay in each sample, and the degeneration degree of the target discs were evaluated using T2‐weighted sagittal magnetic resonance imaging (MRI) according to the Miyazaki disc degeneration grading system. Whether the differences among the three groups were statistically significant was tested using one‐way analysis of variance and an unpaired *t*‐test, respectively.

**Results:**

The differences of the baseline characteristics were not statistically significant between the discogenic neck pain group and the symptomatic control group (*p* > 0.05). The expression of inflammatory cytokines in disc samples from the discogenic neck pain group (NO: 9.89 ± 1.75, IL‐1β: 10.74 ± 1.92, IL‐6:31.65 ± 2.46, and TNF‐α: 5.96 ± 1.91) was increased in comparison with the disc samples from both the symptomatic control group (NO: 7.15 ± 2.78, IL‐1β: 8.03 ± 1.87, IL‐6: 25.79 ± 2.12, and TNF‐α: 4.18 ± 2.87) and the normal control group (NO: 6.11 ± 1.37, IL‐1β: 5.84 ± 2.25, IL‐6: 20.65 ± 1.26, and TNF‐α: 2.05 ± 0.58). The differences were statistically significant (*p* < 0.001). Further, there were no statistical differences in the degree of degeneration between discogenic neck pain group and symptomatic control group.

**Conclusions:**

The increased expression of inflammatory cytokines in diseased cervical intervertebral discs might play a key role in the pathogenesis of discogenic neck pain. Although inflammation is involved in intervertebral disc degeneration, there is no linear positive correlation between the concentration of inflammatory cytokines and the degree of disc degeneration.

## Graphical abstract

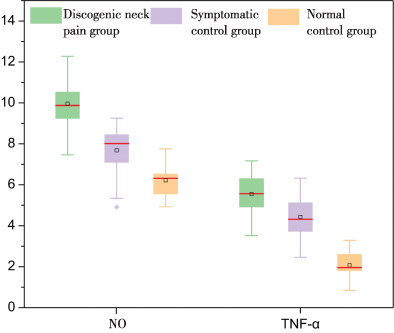

## Introduction

Neck pain is frequently encountered in clinical practice, affecting approximately 66% of adults in their lifetime, while 14% of adults develop chronic neck pain. Although neck pain has become a serious economic and social problem, the etiology remains poorly understood.^1^, ^2^ It has been reported that several cervical disorders might cause neck pain, such as neck muscle tension or strain, minor arthropathy, trauma, cervical disc herniation, abnormal spine curvature, and cervical disc degeneration.^3^ Although there is no direct evidence that degenerative cervical discs are a source of chronic neck pain, it is reasonable to believe that this is true for some patients based on previous studies and clinical practice. It is often found that neck pain is significantly relieved or even resolved following the resolution of neurological symptoms in patients with cervical spondylotic radiculopathy or myelopathy after undergoing anterior cervical discectomy and fusion (ACDF) or artificial cervical intervertebral disc replacement.^4^ Additionally, neck pain can be effectively relieved by performing an intervertebral disc block test on patients suffering from chronic neck pain.^5^ Therefore, it is reasonable to conclude that degenerated cervical discs are a source of chronic neck pain. Neck pain related to degenerative intervertebral discs can usually be divided into two types:^6^ radiation pain or discogenic pain. Radiation pain is caused by spinal stenosis or cervical radiculopathy. Conversely, discogenic pain is caused by disorders of the intervertebral discs and is usually accompanied by neck stiffness, headache, unilateral or bilateral shoulder pain, non‐radicular upper arm pain, ocular and vestibular dysfunction, and anterior chest wall pain,^7^ accounting for a large proportion of chronic neck pain of 16%–41% incidence.^2^, ^5^

Scholars once thought that the intervertebral disc was not innervated; therefore, the diagnosis of discogenic pain was controversial. However, recent studies have shown that intervertebral discs, particularly degenerative ones, have extensive innervation.^8^ In 1988, Bogduk *et al*. described the innervation patterns of the sinuvertebral nerves into the outer third of the annulus fibrosis based on microdissection and histologic studies from clinical patients and cadavers.^10^ Their research also demonstrated the presence of nerve fibers and nerve endings within the outer third of the annulus fibrosis using cholinesterase staining. Moreover, subsequent studies have reported the presence of free nerve endings within human degenerative cervical intervertebral discs.^9^, ^10^, ^11^, ^12^ Substance P and calcitonin gene‐related peptides have also been found in painful lumbar and cervical discs in humans.^13^, ^14^ These studies provide an important anatomical basis for the generation and conduction of discogenic neck pain.

The ingrowth of nociceptive nerve fibers into inner parts of the annulus and even nucleus enables the occurrence of discogenic pain, but not all degenerative discs cause pain. The differences in the expression of inflammatory cytokines might be one of the reasons. Previous research has shown that inflammation is involved in intervertebral disc degeneration,^15^, ^16^, ^17^ and the increased expression of inflammatory cytokines is the prominent feature of degenerative discs.^11^, ^18^, ^19^ These increased cytokines can promote matrix degradation, chemokine production, and changes in cell phenotype. The resulting imbalance between catabolic and anabolic responses leads to degeneration. As main frontier cytokines, nitric oxide (NO), interleukin (IL)‐1, interleukin (IL)‐6, and tumor necrosis factor alpha (TNF‐α) play key roles in the occurrence and progression of intervertebral disc degeneration and discogenic low back pain.^20^ However, the possibility of a similar mechanism of pathogenesis existing in discogenic neck pain has not been previously explored. Therefore, the aims of this study are as follows: (i) determine the expression of inflammatory cytokines in both diseased cervical discs and normal control discs; (ii) explore the possible pathogenesis of discogenic neck pain by analyzing the relationship between inflammatory cytokines and discogenic neck pain; and (iii) provide a valuable reference for the prevention and treatment of discogenic neck pain.

## Methods

### Patients

Samples of cervical discs from cervical spondylotic myelopathy patients with discogenic neck pain were obtained between October 1, 2021, and October 1, 2022.

The inclusion criteria included: (i) severe chronic neck pain (visual analog scale [VAS] ≥70 mm); (ii) ≤2 disease segments; (iii) nonresponse to conservative therapy over a minimum of 3 months; and (iv) no history of cervical trauma, surgery, tuberculosis, infections, tumors, or any demonstrable abnormalities of the congenital or developmental cervical spine. Discogenic neck pain was determined by a positive response to an analgesic discography test.^21^

During the analgesic discography test, no contrast medium was used. If two discs were suspected, the second disc was tested after any effects on the first disc had ceased. While in the supine position, under the guidance of a C‐armed X‐wire machine, a 22‐G spinal needle was percutaneously punctured into the center of the disc via an anterolateral approach, as judged from anteroposterior and lateral views. A small volume (0.3–0.5 mL) of 0.25% bupivacaine was injected. If there was no complaint of discomfort after lying supine for 2 h, the patient was allowed to get up and move around without limiting cervical spine movement. Pain relief of ≥70 percentage was regarded as a positive. Relief percent was calculated using:

$$\begin{array}{l} \left[\left(\text{preoperative}\;\mathrm{VAS}–\text{postoperative}\;\mathrm{VAS}\right)/\text{preoperative}\;\mathrm{VAS}\right]\\ \;\times 100\%. \end{array}$$

Of the 41 patients tested, 28 had a positive response. One patient could not tolerate the surgery due to severe coronary disease; however, the remaining 27 patients underwent anterior cervical surgery using anterior cervical cortectomy and fusion (ACCF) or ACDF surgical procedures (8 and 19 underwent ACCF and ACDF, respectively), during which 38 cervical disc samples were obtained, as seen in the discogenic neck pain group. There were 15 men and 12 women in this group, and the mean age was 57.6 years (range: 37–82 years). The mean duration of neck pain was 13.6 months (range: 3–29 months). The involved surgical levels in the current study were from C3/4 to C6/7.

Simultaneously, we collected 41 cervical disc samples from 29 patients who suffered cervical spondylotic myelopathy without neck pain or with only mild neck pain (VAS ≤30 mm) and who had undergone ACDF or ACCF as a symptomatic control group (11 and 18 underwent ACCF and ACDF, respectively). A total of 14 men and 15 women were recruited in this group, with a mean age of 55.8 years (range: 33–86 years). The surgical levels involved in the current study were also from C3/4 to C6/7. The inclusion criteria for the symptomatic control group were the same as those for discogenic neck pain group, except for the varying degree of neck pain.

Additionally, 32 disc samples were collected from eight fresh cadavers as a normal control group. Five men and three women were in this group, with a mean age of 54.3 years (range: 24–65 years). Before collecting samples, the discs showed no signs of degeneration in the imaging examination.

Before the surgery, two experienced radiologists and two senior spine surgeons were invited to evaluate the degeneration degree of the target discs using T2‐weighted sagittal MRI (3.0T, Philips) according to the Miyazaki disc degeneration grading system.^22^ A detailed record was made, and the procedure was performed under double‐blind conditions. The MRI scan (T2W1, TR 2000 ms and TE 90 ms) layer was 4 mm thick, 1 mm apart, and the matrix was 320 × 192 mm^2^. Discs were scored according to the cervical intervertebral disc degeneration grading system (grades 1–5 of ascending severity), and the score corresponded with the severity grade of the degree of intervertebral disc degeneration. For example, grade 3 corresponds to 3 points, and grade 4 to 4 points. The average of the four scores determined by the two radiologists and two spinologists was taken and regarded as the final disc score.

Written informed consent from each patient was obtained before the research, and the protocol was approved by the Ethics Committee of our hospital (approval number: 202110E). The study was carried out in accordance with relevant guidelines and regulations of the Declaration of Helsinki.

### Expression of Inflammatory Cytokines

The 111 disc samples (38, 41, and 32 in the discogenic neck pain group, the symptomatic control group, and the normal control group, respectively) were stored at −80°C. Samples were repeatedly rinsed with normal saline and dried by blotting with absorbent paper to accurately weigh each tissue sample. Normal saline was added at a weight‐to‐volume ratio of 1:4. The discs from each group were then crushed using a Soniprep150 (Sanyo, UK) ultra‐medium ultrasonic pulverizer, which produced a 20% tissue homogenate by treating the sample at an amplitude of 14 μm for 30 s. The homogenate was then centrifuged at 3000 rpm (4°C) for 15 min. The supernatant was carefully collected and stored in a refrigerator at 6°C. Assays were completed within 48 h.

The expression of NO in each sample was determined using nitrate reductase to convert NO to nitrite (NO_2_^−^), as NO is chemically active and rapidly metabolizes to NO_2_^−^ and nitrate (NO_3_^−^) *in vivo*. The result was determined based on the color development depth. We used the standard curve method and strictly adhered to the instructions in the NO test kit offered by Shanghai Xuan Ya Biotechnology (Shanghai, China). The amount of NO_2_^−^/NO_3_^−^ represented the concentration of NO in the sample expressed as μmol/g protein.

The expression of IL‐1β, IL‐6, and TNF‐α was subsequently measured. The samples were thawed at room temperature in accordance with the operating instructions of the enzyme‐linked immunosorbent assay (ELISA) kits (Shanghai Xuan Ya Biotechnology, Shanghai, China). The concentration of IL‐1β, IL‐6, and TNF‐α in each sample was calculated and recorded using a standard curve and expressed in units of pg./g.

### Statistical Analysis

Statistical analysis of the data was performed using IBM SPSS Statistics version 20.0 (IBM, Armonk, N.Y., USA). The baseline data for age, sex, and the degree of disc degeneration were tested using one‐way analysis of variance (*post hoc*, least significance difference [LSD]), Pearson χ^2^, and unpaired *t*‐test, respectively. The measurement data (concentration of inflammatory cytokines) were analyzed using one‐way analysis of variance (*post hoc*, LSD) among the three groups. A *p*‐value <0.05 was considered statistically significant.

## Results

### Baseline Characteristics

A total of 111 disc samples were examined in this research. The baseline characteristics of all participants are shown in Table 1.

**TABLE 1:** Baseline characteristics of the 56 patients and eight donors in the three groups.

Characteristics	Discogenic neck pain group	Symptomatic control group	Normal control group	*F*‐values	*p*‐values		
					A/B	B/C	A/C
Age (x̄ ± *s, years*)	57.6 ± 11.92	55.8 ± 13.32	54.3 ± 9.71	0.277	0.545	0.810	0.558
Female	12 (44.4)	15 (51.7)	3 (37.5)	0.173	0.733		
Male	15 (55.6)	14 (48.3)	5 (62.5)				
Degree of degeneration	4.3 ± 0.63	4.1 ± 0.67	‐	1.624	0.176		
Level of disc							
C3/4	2 (5.3)	1 (2.4)	8 (25)				
C4/5	12 (31.5)	16 (39.1)	8 (25)				
C5/6	15 (39.5)	19 (46.3)	8 (25)				
C6/7	9 (23.7)	5 (12.2)	8 (25)				

There were no statistically significant differences in the demographic or clinical features (one‐way analysis of variance, *post hoc*, LSD; P of age = 0.545, 0.810, and 0.558 between the discogenic neck pain group and the symptomatic control group, the symptomatic control group and the normal control group, and the discogenic neck pain group and the normal control group, respectively) among the three groups. Significant sex differences were not observed among the three groups (Pearson χ^2^, *p* = 0.733), and no significant differences in the degree of degeneration of the disc samples were found between the discogenic neck pain group and the symptomatic control group (unpaired *t*‐test; *p* = 0.176). Further, the frequency of the four involved segments was similar between the two groups.

### Measurement of Inflammatory Cytokines

According to the ELISA test kit results, the concentration of inflammatory cytokines in the normal control group was the lowest among the three groups. The concentration in the symptomatic control group was greater than that of the normal control group but less than that of discogenic neck pain group. Among the three groups, the concentration of inflammatory cytokines in disc samples from the discogenic neck pain group was the greatest. Differences between the discogenic neck pain group and symptomatic control group, the discogenic neck pain group and normal control group, and the symptomatic control group and normal control group were statistically significant (*p* < 0.001 for all). The concentration of inflammatory cytokines is detailed in Table 2.

**TABLE 2:** Expression of inflammatory cytokines in the disc samples from three groups (x̄ ± *s*).

	Discogenic neck pain group	Symptomatic control group	Normal control group	*F*‐values	*p*‐values		
					A/B	B/C	A/C
NO	9.89 ± 1.75	7.15 ± 2.78	6.11 ± 1.37	30.86	*p* < 0.001	*p* < 0.001	*p* < 0.001
IL‐1β	10.74 ± 1.92	8.03 ± 1.87	5.84 ± 2.25	52.70	*p* < 0.001	*p* < 0.001	*p* < 0.001
IL‐6	31.65 ± 2.46	25.79 ± 2.12	20.65 ± 1.26	252.75	*p* < 0.001	*p* < 0.001	*p* < 0.001
TNF‐α	5.96 ± 1.91	4.18 ± 2.87	2.05 ± 0.58	30.20	*p* < 0.001	*p* < 0.001	*p* < 0.001

*Note*: The data show the concentration of inflammatory cytokines in the cervical disc samples from patients in three groups.

Abbreviations: IL, interleukin; NO, nitric oxide; TNF‐α, tumor necrosis factor alpha.

**FIGURE 1:**
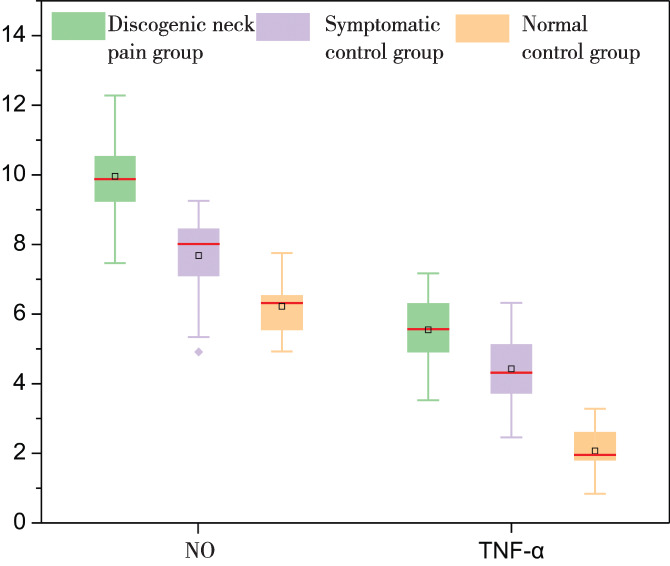
The box plot shows the distribution characteristics of the data of NO and TNF‐α in the discogenic neck pain group, the symptomatic control group, and the normal control group. The small square inside the box indicates the mean value. The red line inside the box indicates the median. The colored spot outside the box shows the outliers. Unit of NO: μmol/g protein. Unit of TNF‐α: pg./g. NO, nitric oxide; TNF‐α, tumor necrosis factor alpha.

**FIGURE 2:**
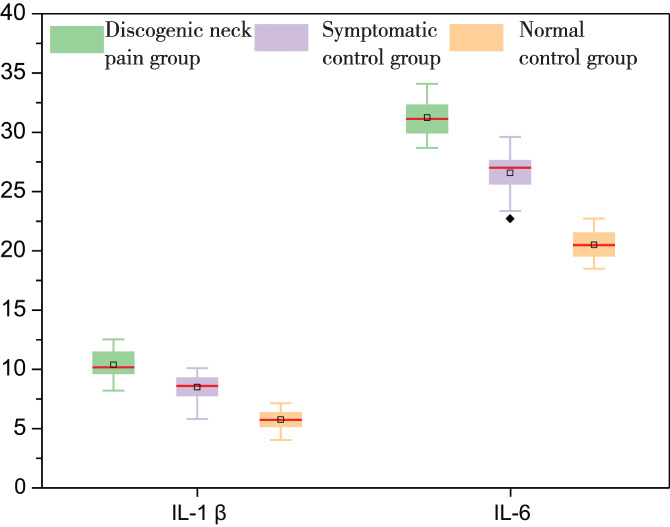
The box plot shows the distribution characteristics of the data of IL‐1β and IL‐6 in the discogenic neck pain group, the symptomatic control group, and the normal control group. The small square inside the box shows the mean value. The red line inside the box shows the median. The black spot outside the box shows the outliers. Unit of IL‐1β and IL‐6: pg./g. IL, interleukin.

In addition, we analyzed the original data of the concentration of inflammatory cytokines using Origin Pro 2023 (The Ultimate Software for Graphing and Analysis, OriginLab). Figure 1 shows the distribution of the data of NO and TNF‐α among the three groups. The mean, median, and maximal value of NO are the greatest in group A and lowest in group C. The data distribution of TNF‐α among the three groups shows the same trend. Figure 2 shows the same trend in the distribution of the data of IL‐1β and IL‐6 among the three groups.

## Discussion

Chronic neck pain, similar to chronic low back pain, has become a severe public health problem worldwide.^1^, ^5^ Research in this field has always been the focus of scholars due to the limited understanding of its etiology.

The degenerative cervical intervertebral disc, an important component of the physiological structure of the cervical spine, has been established as a main source of cervical pain.^9^, ^11^ According to the results of this research, the expression of inflammatory cytokines in cervical disc samples from patients in the discogenic neck pain group increased significantly compared to that in the symptomatic control group. The two groups have no statistical differences in baseline characteristics; therefore, it is reasonable for us to conclude that the increased expression of inflammatory cytokines might play a key role in the pathomechanism of discogenic neck pain. Further, it has been reported that ingrowth of nociceptive receptors into diseased cervical intervertebral disc is associated with discogenic neck pain.^14^ According to the results of current and previous research, it is reasonable to conclude that an increased expression of inflammatory cytokines in diseased cervical discs might interact with abnormally in‐growing nerve endings, substance P, and calcitonin gene‐related peptides to produce an amplification called “peripheral sensitization” for mechanical stimuli and compressive stress that are below normal thresholds. Eventually, this leads to the occurrence of discogenic neck pain.^9^

### Exploration of Pathogenesis of Discogenic Neck Pain

A similar viewpoint can also be found in previous studies focusing on discogenic low back pain. Recently, Peng *et al*., Wu *et al*., and Yang *et al*. reported that the expression of inflammatory cytokines in painful cervical discs was increased.^4^, ^11^, ^14^ However, because the conclusion was drawn from a comparison with normal disc samples, interference from the relationship between inflammation and disc degeneration could not be excluded effectively. We compared the expression of inflammatory cytokines in cervical disc samples from the discogenic neck pain group and the symptomatic control group. The conclusions drawn from this study are more direct and reliable because there is no statistical difference in the degree of degeneration between the two groups. Such a comparison has not been reported in previous studies. In addition, no statistical difference in the degree of degeneration has been found between the discogenic neck pain group and the symptomatic control group, which might indicate that there is no linear positive correlation between the concentration of inflammatory cytokines and the degree of disc degeneration. Moreover, the expression of inflammatory cytokines in the diseased cervical discs in both the discogenic neck pain group and symptomatic control group is greater than that in the normal control group. This might additionally confirm the conclusion that inflammation is involved in the process of intervertebral disc degeneration.^19^, ^23^, ^24^, ^25^

Increased expression of inflammatory cytokines, such as NO, TNF‐α, IL‐1, IL‐2, IL‐4, IL‐6, IL‐8, IL‐10, and interferon γ, are prominent features of degenerative discs that can promote the degradation of the extracellular matrix, the release of chemokines, and alteration of the cellular phenotype.^20^ Interactively, the released chemokines can promote the infiltration and activation of T cells, B cells, and macrophages, further amplifying the inflammatory cascade and promoting the release of neurotrophins, particularly nerve growth factor. Moreover, the inflammatory state within the degenerative disc triggers multiple pathogenic responses, such as cellular senescence and apoptosis, ingrowth of nerves and blood vessels, and discogenic pain.^24^, ^26^ This is consistent with the results of the current study; however, the occurrence of discogenic neck pain is a very complex pathological process, and its exact pathomechanism requires further in‐depth study. Whether there is a potential link between the degree of disc degeneration and discogenic pain remains inconclusive because not all degenerative intervertebral discs can cause pain. Furthermore, although we found that the expression of inflammatory cytokines is increased in painful discs, the increased expression of inflammatory cytokines does not always cause pain. For example, the expression of inflammatory cytokines in the cervical disc samples of the symptomatic control group was greater than that in the normal control group. However, the patients in this group predominantly had mild neck pain or no neck pain. If there is a threshold of the occurrence of neck pain, the value of this threshold is worth studying.

### Controversy and Dissension

Similar to discogenic low back pain, the diagnosis of discogenic neck pain is also debatable, as there is no standardized diagnostic method established. Use of discography, the only test that links the disease with clinical symptoms, remains controversial.^2^ This dissension is primarily due to two aspects: (i) false positives are inevitable as increased pressure might induce pain within a normal intervertebral disc^5^; and (ii) degeneration might be induced or accelerated in normal intervertebral discs due to a puncture injury. Discography is usually only conducted on discs that show degenerative signs on imaging. To ensure surgical effects, discography is conducted before surgery; the discs showing normal signs on imaging are not usually referred for discography.^4^ Because the discography needle is 22G, the puncture injury is negligible.

In current research, analgesic discography has been adopted to locate the painful‐disc. Although false‐negative responses are indeed inevitable as local anesthetic cannot fully anesthetize all sources of pain, this might only cause some deviation in the sample size of the discogenic neck pain group and does not affect the results of the current study because patients with negative results have been excluded. Moreover, the suspected discs were injected with only 0.3–0.5 mL bupivacaine. Even if a small amount of bupivacaine leaked out of the intervertebral disc, it could not have reached the posterior facet joints, nor could it have produced an anesthetic effect in the cervical nerve roots. Therefore, any false‐positive responses can be effectively excluded.

It is well known that inflammatory cytokines are released by living cells, so the ideal disc samples in the normal control group should be collected from healthy humans. However, this source pathway is limited and is also forbidden by the code of ethics. To minimize bias, eight fresh frozen cadavers were used in the current study. Although the detected concentration of inflammatory cytokines might be different from the actual data, this would not interfere with the study conclusions because the main conclusions of this research were drawn from a comparison between the discogenic neck pain group and symptomatic control group. The conclusion that inflammation is involved in the process of intervertebral disc degeneration, which was drawn from the comparison that included the participation of the normal control group, has been extensively demonstrated in previous studies.

### Limitations and Strengths

The present study has several limitations. First, we determined the expression of inflammatory cytokines in each sample using ELISA test kits that process the whole intervertebral disc into a tissue homogenate. The results would be more robust if the disc could be tested separately according to the source site, such as the outer layer, inner layer, or nucleus pulposus. Second, it was difficult to determine if the mild neck pain associated with the symptomatic control group was discogenic. Moreover, given that pain is a subjective feeling, pain thresholds vary across different individuals; therefore, whether these factors have caused bias in the results remains unclear. Additionally, the sample size in this study was small; studies with larger sample sizes are warranted to verify these conclusions.

In addition, several strengths of current research need to be noted. First, analgesic discography has been adopted to diagnose discogenic neck pain, as it is the most effective test for diagnosis and location in painful‐disc syndrome.^21^ It can not only overcome the disadvantages of conventional discography, such as the high false positive rate resulting from the lack of specific imaging signs and pain style but also identify the disc responsible for pain and aid in localizing the diagnosis. Second, the main conclusions were drawn from the comparison between the discogenic neck pain group and the symptomatic control group. There were no statistical differences in baseline characteristics between the two groups, so the conclusions were more reliable.

## Conclusion

In conclusion, based on our current research results, we infer that the increased expression of inflammatory cytokines in diseased cervical intervertebral discs plays a key role in the pathogenesis of discogenic neck pain. Although inflammation is involved in intervertebral disc degeneration, there is no positive linear correlation between the concentration of inflammatory cytokines and the degree of disc degeneration. However, due to the complexity of discogenic neck pain, its detailed pathogenesis needs to be further studied.

## Author Contributions

Conceptualization, Xinjian Kang, and Man Qian; methodology, Tao Qin, Mingle Liu; software, Huawei Xu; validation, Xinjian Kang, Tao Qin, and Baoshan Xu; formal analysis, Man Qian; investigation, Xinjian Kang, Mingle Liu; resources, Huawei Xu; data curation, Xinjian Kang; writing—original draft preparation, Xinjian Kang; writing—review and editing, Man Qian; visualization, Tao Qin; supervision, Baoshan Xu; project administration, Baoshan Xu; funding acquisition, not applicable. All authors have read and agreed to the published version of the manuscript.

## Conflict of Interest

The authors declare that there is no conflict of interest regarding the publication of this article.

## Funding Statement

This research received no external funding.

## Authorship Declaration

All authors listed meet Editors and are in agreement with the manuscript. the authorship criteria according to the latest guidelines of the International Committee of Medical Journal.

## Ethical Statement

This study was approved by the Ethics Committee of Traditional Chinese Medicine Hospital of Qinhuangdao (approval number: 202110E). Informed consent was obtained from all study participants, and the study was carried out in accordance with relevant guidelines and regulations of the Declaration of Helsinki.

## Data Availability

The datasets used during the current study available from the corresponding author on reasonable request.
